# Electronic and Structural Transitions of LaAlO_3_/SrTiO_3_ Heterostructure Driven by Polar Field‐Assisted Oxygen Vacancy Formation at the Surface

**DOI:** 10.1002/advs.202103095

**Published:** 2021-09-08

**Authors:** Kyung Song, Taewon Min, Jinsol Seo, Sangwoo Ryu, Hyungwoo Lee, Zhipeng Wang, Si‐Young Choi, Jaekwang Lee, Chang‐Beom Eom, Sang Ho Oh

*Adv. Sci*. **2021**, *8*, 2002073

DOI: 10.1002/advs.202002073

In the originally published article, one funding information is missing in the acknowledgments. Please find the correct acknowledgements here:

